# Comparative efficacy of modified antagonist protocols combining clomiphene citrate or letrozole for ovarian stimulation in patients with normal ovarian reserve

**DOI:** 10.3389/fendo.2026.1814317

**Published:** 2026-04-28

**Authors:** Huijuan Wei, Huilan Lin, Liyi Cai, Weimin Yang, Xiaoxi Li, Xiaoyan Wang, Chenyi Wang, Jing Wang, Qingqing Cheng, Kai Deng

**Affiliations:** 1 Department of Reproductive Medicine, Hebei Maternity Hospital, Shijiazhuang, China; 2 Shi Jiazhuang Technology Innovation Center of Precision Prevention and Control of Birth Defects, Shijiazhuang, China; 3 Department of Cell Biology, School of Basic Medicine, Hebei Medical University, Shijiazhuang, China; 4 School of Biomedical Engineering, Hubei University of Medicine, Shiyan, China

**Keywords:** antagonist protocol, clomiphene citrate, *in vitro* fertilization, letrozole, normal ovarian reserve, ovarian hyperstimulation syndrome

## Abstract

**Background:**

To compare the efficacy of modified gonadotropin-releasing hormone (GnRH) antagonist protocols combining clomiphene citrate (CC) or letrozole (LE) versus a conventional antagonist protocol in patients with normal ovarian reserve, aiming to reduce gonadotropin (Gn) consumption and ovarian hyperstimulation syndrome (OHSS) risk.

**Methods:**

This retrospective cohort study included 565 *in-vitro* fertilization/intracytoplasmic sperm injection (IVF/ICSI) cycles. Patients were divided into three groups based on the stimulation protocol: CC group (*n* = 217; CC 50 mg/day throughout stimulation), LE group (*n* = 148; LE 2.5 mg/day for 5 days), and Control group (*n* = 200; conventional antagonist protocol). The primary outcome was clinical pregnancy rate (CPR). Secondary outcomes included live birth rate, implantation rate, miscarriage rate, and laboratory parameters.

**Results:**

Baseline characteristics were similar among groups. Both CC and LE groups required significantly shorter Gn duration and lower total Gn dose than the control group (both *P* < 0.001). GnRH antagonist consumption was lowest in the LE group (*P* < 0.001). On the trigger day, estradiol (E2) levels were significantly lower in the LE group than in the CC and control groups (*P* < 0.001). Although the LE group was associated with a lower oocyte yield than the control group (*P* < 0.05), it had a significantly higher day-3 top-quality embryo rate (52.9% vs. 44.1%, *P* < 0.001). The CC group exhibited a higher cycle-specific top-quality embryo rate after blastocyst culture (55.7% vs. 45.9%, *P* < 0.001). In fresh transfer cycles, CPR was comparable between the LE and control groups (60.0% vs. 48.3%, *P* = 0.172). Live birth rates were comparable among the three groups in FET cycles (40.2%, 40.2%, and 45.2%, respectively; *P* = 0.709). In frozen-thawed embryo transfer (FET) cycles, no significant differences were observed in CPR, implantation rate, miscarriage rate, or live birth rate among the three groups.

**Conclusions:**

Modified GnRH antagonist protocols with CC or LE were associated with reduced Gn and antagonist consumption and decrease oocyte yield but were associated with higher embryo quality metrics and achieve comparable clinical pregnancy outcomes in normal ovarian reserve patients. These protocols are associated with reduced medication costs to conventional stimulation.

## Introduction

The number of patients undergoing *in-vitro* fertilization/intracytoplasmic sperm injection-embryo transfer (IVF/ICSI-ET) is increasing, and most of them have normal ovarian reserve. Controlled ovarian stimulation (COS) is a critical determinant of treatment success. The conventional long protocol offers advantages such as high oocyte yield and pregnancy rates, but it is associated with substantial costs, prolonged treatment duration, and an increased risk of ovarian hyperstimulation syndrome (OHSS) (1, 2). The gonadotropin-releasing hormone (GnRH) antagonist protocol overcomes several limitations of the long protocol, reducing medication exposure and OHSS incidence (3, 4).

Clomiphene citrate (CC) and letrozole (LE) are oral ovulatory agents that can be integrated into COS protocols. CC, a selective estrogen receptor modulator, is commonly used in mild stimulation protocols for predicted poor responders (5–9). LE, an aromatase inhibitor, reduces estrogen levels and may improve follicular response (10). However, evidence regarding their use in combination with antagonist protocols for patients with normal ovarian reserve remains limited. This study aims to compare the clinical efficacy and laboratory outcomes of conventional antagonist protocols with modified protocols incorporating full-course CC or LE in this patient population to provide evidence for safer and more cost-reduction treatment strategies.

## Materials and methods

### Study design and population

This retrospective cohort study analyzed 565 IVF/ICSI cycles performed at the Department of Reproductive Medicine, Hebei Maternity Hospital, between July 2020 and March 2022. Participants were divided into three groups based on their COS protocol: the CC group (*n* = 217), the LE group (*n* = 148), and the conventional antagonist protocol control group (*n* = 200).

Inclusion criteria were ① female age ≤ 37 years; ② normal ovarian reserve, defined as anti-Müllerian hormone (AMH) between 1.0 and 4.0 ng/ml and antral follicle count (AFC) between 6 and 15; ③ basal follicle-stimulating hormone (FSH) < 10 mIU/ml; and ④ no history of cycle cancellation due to hyper- or hypo-ovarian response. Exclusion criteria included: endocrine disorders (e.g., polycystic ovary syndrome, hyperprolactinemia), recurrent pregnancy loss, endometriosis, adenomyosis, uterine anomalies, intrauterine adhesions, thin endometrium, or other autoimmune diseases.

The study was approved by the Institutional Review Board of Hebei Maternity Hospital (Approval Number: 20210002), and all participants provided written informed consent.

### Ovarian stimulation protocols

CC group: Oral CC (50 mg/day) was administered from menstrual cycle days 2 to 3 until the day of trigger. Human menopausal Gn (HMG; Livzon Group) injections (150–300 IU/day) commenced concurrently from days 2 to 3. GnRH antagonist (Cetrotide, Merck Serono; 0.125–0.25 mg/day) was introduced when a leading follicle reached 12 mm or serum LH showed an increasing trend.

LE group: Oral LE (2.5 mg/day) was administered from menstrual cycle days 2 to 3 for 5 days. Subsequent stimulation with HMG and GnRH antagonist addition followed the same protocol as the CC group.

Control group: Recombinant FSH (GONAL-f, Merck Serono; 150–300 IU/day) combined with HMG was administered subcutaneously from menstrual cycle days 2 to 3. GnRH antagonist was added as in the other groups.

In all groups, recombinant FSH was supplemented if necessary. Final oocyte maturation was triggered with a dual trigger (0.1 mg GnRH agonist [triptorelin, Pfizer] + 4000 IU hCG [Livzon Group]) when at least 2–3 dominant follicles reached 18 mm in diameter. Oocyte retrieval was performed 36–37h later.

### Embryo culture, transfer, and luteal support

In the LE and Control groups, fresh embryo transfer was performed if endometrial thickness was ≥8 mm, no OHSS risk was present, and progesterone (P) level was <2 ng/ml. Luteal support was provided with daily intramuscular progesterone (40 mg) starting on the retrieval day and oral estradiol valerate (3 mg twice daily) from the following day. One or two cleavage-stage embryos were transferred on day 3. Surplus viable embryos were cultured to blastocysts for vitrification.

In the CC group, all embryos were cryopreserved by protocol design due to the known anti-estrogenic effect of CC on the endometrium, and frozen-thawed embryo transfer (FET) was performed one to two menstrual cycles later. For patients in other groups ineligible for fresh transfer, all embryos were cryopreserved (either at the cleavage stage or after blastocyst culture) for subsequent FET cycles.

It is important to note that, by protocol design, all patients in the CC group underwent a freeze-all strategy due to the anti-estrogenic effect of CC on the endometrium, whereas patients in the LE and control groups received fresh transfers when clinically eligible. This systematic difference in treatment pathways limits direct comparability of clinical outcomes across groups.

### Outcome measures

The primary outcome was the clinical pregnancy rate (CPR), defined as the visualization of a gestational sac on ultrasound 21–35 days after transfer. Secondary outcomes included implantation rate, miscarriage rate, live birth rate, laboratory parameters (number of oocytes retrieved, 2PN fertilization rate, number of D3 usable embryos, D3 top-quality embryo rate, blastocyst formation rate, top-quality blastocyst rate, total transferable embryos, and cycle-specific top-quality embryo rate), and stimulation parameters (duration of Gn, total Gn dosage, GnRH antagonist dosage, E2 and LH levels on trigger day, and endometrial thickness).

### Statistical analysis

Statistical analyses were performed using SPSS version 23.0 (IBM Corp.). Continuous variables with normal distribution are presented as mean ± standard deviation (SD) and were compared using one-way ANOVA with *post-hoc* LSD or Tamhane’s T2 tests, as appropriate. Categorical variables are presented as numbers (percentages) and were compared using the chi-square test. A *P*-value of <0.05 was considered statistically significant.

## Results

### Baseline characteristics

As shown in **Table 1**, there were no significant differences among the three groups in female age, BMI, duration of infertility, AMH levels, AFC, or basal FSH levels (*P* > 0.05), indicating comparable baseline characteristics.

**Table 1:** Baseline characteristics of the study participants (Mean ± SD).

Characteristic	CC group (*n* = 217)	LE group (*n* = 148)	Control group (*n* = 200)	*P*-value
Age (years)	32.01 ± 3.48	31.28 ± 3.37	31.63 ± 3.25	0.122
Infertility duration (years)	3.77 ± 3.19	4.03 ± 2.98	3.33 ± 2.35	0.066
BMI(kg/m2)	24.03 ± 4.19	24.09 ± 4.38	23.68 ± 3.59	0.573
AMH (ng/ml)	2.35 ± 0.88	2.26 ± 0.78	2.46 ± 0.87	0.092
AFC (n)	11.46 ± 3.26	11.32 ± 3.15	11.84 ± 2.84	0.250
Basal FSH (mIU/ml)	8.29 ± 2.96	8.11 ± 2.37	7.73 ± 2.36	0.087

Values are presented as mean ± SD. *P*-values were calculated using one-way ANOVA.

### Stimulation parameters

Stimulation outcomes are detailed in **Table 2**. The CC and LE groups had significantly shorter stimulation durations and lower total Gn doses compared to the control group (*P* < 0.001). The LE group required the least amount of GnRH antagonist, followed by the CC group, with the control group requiring the most (*P* < 0.001). Endometrial thickness on the day of the hCG trigger was significantly thinner in the CC group compared to the LE and control groups and thinner in the LE group compared to the control group (*P* < 0.001). The LE group had significantly lower E2 levels on trigger day than both the CC and control groups (*P* < 0.001). Conversely, LH levels on trigger day were highest in the LE group, intermediate in the CC group, and lowest in the control group (*P* < 0.001).

**Table 2:** Comparison of stimulation parameters among the three groups (Mean ± SD).

Parameter	CC group (*n* = 217)	LE group (*n* = 148)	Control group (*n* = 200)	*P-*value
Gn duration (days)	9.52 ± 1.53^*^	9.38 ± 1.57^*^	10.32 ± 1.67	< 0.001
Total Gn dose (IU)	1981.52 ± 577.27^*^	1943.92 ± 485.54^*^	2245.81 ± 548.14	< 0.001
GnRH antagonist dose (mg)	0.69 ± 0.36^*,#^	0.60 ± 0.31^*^	0.78 ± 0.39	< 0.001
E2 on hCG day (pg/ml)	3043.08 ± 1467.30	1499.64 ± 1009.12^*^	2876.14 ± 1393.36	< 0.001
LH on hCG day (mIU/ml)	4.01 ± 2.60^*,#^	5.13 ± 3.87^*^	2.62 ± 1.90	< 0.001
Endometrial thickness (mm)	8.56 ± 2.72^*,#^	10.47 ± 2.96^*^	11.12 ± 2.64	< 0.001

*P < 0.05 compared to the Control group; #*P* < 0.05 compared to the LE group.

### Laboratory outcomes

Key laboratory outcomes are presented in **Table 3**. The number of oocytes retrieved and 2PN zygotes was significantly lower in the LE group compared to the CC and control groups (*P* < 0.05). The number of D3 usable embryos was lower in the LE group than in the control group. However, the D3 top-quality embryo rate was significantly higher in the LE group (52.9%) than in both the CC (44.1%) and control (44.1%) groups (*P* < 0.001). The CC and LE groups had fewer total transferable embryos than the Control group, but the CC group exhibited a significantly higher cycle-specific top-quality embryo rate (55.7%) compared to the LE (48.8%) and control (45.9%) groups (*P* < 0.001). No significant differences were observed in the 2PN fertilization rate, blastocyst formation rate, or top-quality blastocyst rate among the three groups.

**Table 3:** Comparison of laboratory outcomes among the three groups.

Parameter	CC group (*n* = 217)	LE group (*n* = 148)	Control group (*n* = 200)	*P-*value
Oocytes yielded (*n*)	10.06 ± 5.33	8.76 ± 4.01^*^	11.06 ± 5.41	< 0.001
2PN number (*n*)	5.89 ± 3.85	4.91 ± 2.91^*^	6.70 ± 4.06	< 0.001
D3 usable embryos (*n*)	4.60 ± 3.40	4.04 ± 2.82^#^	5.15 ± 3.30	0.006
D3 top-quality embryos (*n*)	2.53 ± 2.68	2.52 ± 2.17	2.82 ± 2.44	0.407
Transferable embryos (*n*)	3.89 ± 2.62	3.57 ± 2.43	4.62 ± 2.79^*^	0.001
Cycle-specific top-quality embryos (*n*)	2.17 ± 2.03	1.74 ± 1.88	2.12 ± 2.08	0.108
2PN rate (%)	62.1(1279/2058)	59.5(726/1221)	61.9(1314/2124)	0.270
D3 top-quality embryos rate (%)	44.1(550/1247)	52.9(373/705)^*^	44.1(564/1280)	< 0.001
Blastocyst formation rate (%)	57.3(681/1188)	59.4(342/576)	59.0(676/1146)	0.620
Top-quality blastocyst rate (%)	34.4(234/681)	31.6(108/342)	31.7(214/676)	0.501
Cycle-specific top-quality blastocyst rate (%)	55.7(471/845)^*^	48.8(258/529)	45.9(424/924)	< 0.001

*P < 0.05 compared to the other two groups; #*P* < 0.05 compared to the Control group. Cycle-specific top-quality embryos: final top-quality cleavage-stage embryos + top-quality blastocysts after culture.

### Clinical outcomes

Among the 144 cycles that proceeded to fresh embryo transfer (LE group *n* = 55, control group *n* = 89; others canceled due to heterogeneous endometrium or patient preference), the CPR was higher in the LE group (60.0%) than in the control group (48.3%), though the difference was not statistically significant (*P* = 0.172). The LE group had significantly lower E2 levels on trigger day (**Table 4**). Notably, the implantation rate showed a positive trend in the LE group (43.3% vs. 36.4%, *P* = 0.279).

**Table 4:** Clinical outcomes of fresh embryo transfer cycles between LE and control groups.

Parameter	LE group (*n* = 55)	Control group (*n* = 89)	*P-*value
Age (years)	31.02 ± 3.02	31.65 ± 3.03	0.224
Infertility Duration (years)	4.09 ± 2.93	3.53 ± 2.41	0.213
Endometrium thickness on hCG day (mm)	10.50 ± 2.27	10.83 ± 2.25	0.389
E_2_ on hCG day (pg/ml)	1415.96 ± 965.14#	2531.68 ± 1115.12	< 0.001
Mean number of transferred embryos	1.76 ± 0.43	1.70 ± 0.46	0.387
Clinical pregnancy rate (%)	60.0(33/55)	48.3(43/89)	0.172
Implantation rate(%)	43.3(42/97)	36.4(55/151)	0.279
Miscarriage rate (%)	12.1(4/33)	9.3(4/43)	0.721

#*P* < 0.05 compared to the control group.

In FET cycles, there were no significant differences among the three groups in terms of CPR, implantation rate, or miscarriage rate (*P* > 0.05, **Table 5**). However, the mean number of embryos transferred in the LE group was significantly higher than in the control group (1.50 ± 0.50 vs. 1.30 ± 0.46, *P* < 0.05). Live birth rates were 40.22% (72 of 179) in the CC group, 40.24% (33 of 82) in the LE group, and 45.16% (42 of 93) in the control group. The Pearson chi-square test showed no statistically significant difference among groups [χ²(2) = 0.687, *P* = 0.709]; pairwise comparisons with Bonferroni correction also revealed no significant differences (all adjusted *P* > 0.05).

**Table 5:** Clinical outcomes after frozen-thawed embryo transfer (FET) among groups.

Parameter	CC group (*n* = 179)	LE group (*n* = 82)	Control group (*n* = 93)	*P-*value
Age (years)	31.84 ± 3.61	31.22 ± 3.63	31.55 ± 3.50	0.424
Endometrial thickness on FET day (mm)	9.93 ± 1.83	9.92 ± 2.140	9.78 ± 1.85	0.817
Mean number of transferred embryos	1.39 ± 0.49	1.50 ± 0.50#	1.30 ± 0.46	0.027
Clinical pregnancy rate (%)	58.7(105/179)	56.1(46/82)	61.3(57/93)	0.784
Implantation rate(%)	47.4(118/249)	43.9(54/123)	53.7(65/121)	0.294
Miscarriage rate (%)	15.2(16/105)	15.2(7/46)	14.0(8/57)	0.977
Live birth rate (%)	40.22 (72/179)	40.24 (33/82)	45.16 (42/93)	0.709

#*P* < 0.05 compared to the control group.

## Discussion

This retrospective study demonstrates that combining CC or LE into GnRH antagonist protocols for patients with normal ovarian reserve offers a favorable balance between efficacy, safety, and cost. The key findings are (1) a significant reduction in Gn and antagonist consumption, (2) a lower oocyte yield but improved embryo quality parameters, and (3) comparable clinical pregnancy and live birth rates despite a “freeze-all” strategy in the CC group.

### Cost reduction analysis

The reduction in Gn dosage and duration with CC and LE protocols translates directly into lower medication costs and reduced injection burden for patients, enhancing treatment accessibility and compliance (11–13). Specifically, the total Gn dose was reduced by 264 IU in the CC group and 302 IU in the LE group compared to controls, and the GnRH antagonist dose was reduced by 0.09 mg and 0.18 mg, respectively. Based on current local market prices (recombinant FSH ~¥4.2/IU, HMG ~¥0.27/IU, and antagonist ~¥350/0.25 mg), the LE group saved approximately ¥1200–3000 per cycle compared to the control group, and the CC group also achieved significant cost savings. These data demonstrate the favorable cost reduction of both modified protocols. It is important to emphasize that these estimates represent a descriptive cost reduction analysis, not a formal cost reduction study (e.g., no incremental cost reduction ratio or quality-adjusted life years were calculated).

### Safety and OHSS risk

The significantly lower E2 levels in the LE group and the reduction in the dosage of Gn in both modified protocols suggest a potentially lower risk of OHSS, a critical safety advantage in COS. This is particularly relevant given that even normal responders can develop OHSS, and preventive strategies are paramount. In our study population (normal ovarian responders) with dual trigger, no moderate or severe OHSS occurred, and mild OHSS rates were low across groups. Given the low incidence of OHSS in our study population and the retrospective design, our study lacked sufficient statistical power to detect differences in OHSS rates. Therefore, conclusions regarding the safety advantage of the modified protocols regarding OHSS remain preliminary and hypothesis generating.

### Molecular mechanisms and embryo quality

The observation of higher D3 top-quality embryo rates in the LE group and a higher cycle-specific top-quality embryo rate in the CC group is noteworthy. This suggests that these oral agents may promote more synchronized follicular development or a better hormonal environment for oocyte cytoplasmic maturation, despite retrieving fewer oocytes.

Mechanisms of CC: CC acts as a selective estrogen receptor modulator, competing with estrogen for binding sites in the hypothalamus and pituitary, thereby interfering with estrogen negative feedback and promoting FSH and LH synthesis and release. Additionally, CC may suppress the positive feedback of estrogen on the pituitary, inhibiting LH surges and preventing premature ovulation (14).

Mechanisms of LE: LE is an aromatase inhibitor that binds to the heme group of the cytochrome P450 subunit, inhibiting the conversion of androgens (testosterone and androstenedione) to estrogens (10). This reduces serum estrogen levels, attenuates negative feedback on the hypothalamus, and increases FSH secretion. Simultaneously, intraovarian androgen accumulation enhances FSH receptor expression in granulosa cells, synergistically promoting follicular development (15–20). The intraovarian androgen accumulation induced by LE may enhance follicular sensitivity to FSH, potentially contributing to improved cytoplasmic maturation and subsequent embryo quality.

### LH levels in the LE group

The LE group showed significantly higher LH levels on the trigger day. This is likely due to two factors: (1) the reduced estrogen levels from LE treatment delayed the need for GnRH antagonist initiation and (2) LE’s central effect reduces estrogen−mediated negative feedback, promoting endogenous LH secretion. Recent evidence provides context for this observation: Luo et al. (21) found that in antagonist cycles, LH levels on trigger day were not correlated with outcomes except in specific subgroups; Liu et al. (22) reported that higher LH trajectories were associated with increased oocyte and embryo quantity but not necessarily quality. Therefore, while the higher LH in the LE group may be associated with increased oocyte yield, but this requires further investigation, whether it directly underlies the improved top-quality embryo rate requires further investigation.

### Comparison with recent studies

Our findings align with recent literature. Mandelbaum et al. (14) demonstrated that continuous CC use throughout ovarian stimulation reduced costs, decreased injection burden, and prevented premature ovulation in diminished ovarian reserve patients. Hernandez-Nieto et al. (23) showed that recent CC exposure did not impact subsequent FET outcomes. Grädel et al. (24) reported that low-dose CC did not impair implantation or live birth rates. For LE, Zakerinasab et al. (13) provided meta-analytic evidence supporting LE co-treatment in reducing Gn consumption and maintaining comparable pregnancy outcomes.

### CC group and freeze-all strategy

The compromised endometrial thickness in the CC group, a known anti-estrogenic effect, necessitated a “freeze-all” approach. The subsequent comparable FET outcomes across all groups validate the effectiveness of segmenting the treatment cycle in such scenarios. Recent studies (23, 24) have shown that CC does not have a long-term negative impact on endometrial receptivity or FET outcomes, supporting our protocol design.

Our findings align with emerging concepts in ART that prioritize embryo quality over sheer oocyte quantity, particularly for normal and high responders. The modified protocols presented here are especially suitable for patients with a high AFC or high AMH levels, who are at increased risk of OHSS with conventional stimulation. The comparable CPR in FET cycles across groups, despite fewer oocytes and embryos in the modified protocol groups, underscores the efficiency of these regimens in generating euploid-competent embryos (25).

It should be noted that CPR was the primary outcome of this study. However, given the increasing use of freeze-all strategies and the importance of live birth as the ultimate success measure, we also report live birth rates. The lack of significant differences in live birth rates across groups supports the feasibility of the modified protocols, but the interpretation of CPR as a primary endpoint is limited by potential attrition and the fact that not all clinical pregnancies result in live births.

### Limitations

This study has limitations inherent to its retrospective design. Although baseline characteristics were similar across groups, we did not adjust for potential confounders (e.g., through multivariable regression or propensity score methods) due to the exploratory nature of this analysis. Consequently, unmeasured confounding factors and selection bias cannot be entirely ruled out. Our findings should therefore be interpreted as hypothesis generating, and causal inferences should not be drawn. Future prospective, randomized controlled trials are warranted to confirm these observations.

Notably, the comparison of clinical outcomes among the three groups reflects not only the pharmacological effects of CC or LE but also inherent differences in treatment strategies, specifically, the universal freeze-all approach in the CC group versus the mixed fresh/frozen transfer policy in the other groups. This should be considered when interpreting the comparable pregnancy rates across groups.

The sample size for fresh transfers, particularly in the LE group (*n* = 55), was relatively small, which may have limited the statistical power to detect significant differences in CPRs and live birth rates despite the observed numerical trends. Additionally, the study lacked long-term follow-up data on neonatal outcomes. Future prospective, randomized controlled trials with larger sample sizes are warranted to confirm these findings, and larger studies are needed to evaluate OHSS risk as a primary endpoint.

## Conclusion

For infertile women with normal ovarian reserve, modified GnRH antagonist protocols incorporating CC or LE are cost-reduction alternatives to conventional stimulation. They significantly were associated with reduced Gn and antagonist usage, decreased the number of retrieved oocytes while having higher embryo quality metrics, and ultimately yielded comparable clinical pregnancy outcomes with a potentially lower OHSS risk. These protocols represent a valuable strategic option in the personalized management of ovarian stimulation.

## Data Availability

The original contributions presented in the study are included in the article/supplementary material. Further inquiries can be directed to the corresponding author.
